# Use of health care services according to functional performance in community-dwelling older adults in Spain. An approach using GAMLSS models

**DOI:** 10.1371/journal.pone.0277681

**Published:** 2022-11-17

**Authors:** Maider Mateo-Abad, Kalliopi Vrotsou, María Padilla Ruiz, Alonso Montiel-Luque, María del Carmen Saucedo Figueredo, Mónica Machón, Francisco Rivas Ruiz, Itziar Vergara

**Affiliations:** 1 Instituto de Investigación Sanitaria Biodonostia, Grupo de Atención Primaria, Donostia-San Sebastián, Gipuzkoa, España; 2 Red de Investigación en Cronicidad, Atención Primaria y Promoción de la Salud (RICAPPS), Salamanca, España; 3 Red de Investigación en Servicios Sanitarios en Enfermedades Crónicas (REDISSEC), Marbella, España; 4 Unidad de Investigación, Agencia Sanitaria Costa del Sol, Marbella, Málaga, España; 5 Centro de Salud San Miguel, Distrito Sanitario Costa del Sol, Torremolinos, Málaga, España; 6 Centro de Salud Los Boliches, Distrito Sanitario Costa del Sol, Fuengirola, Málaga, España; University of Auckland, NEW ZEALAND

## Abstract

Functional performance in older adults is a predictor of survival and other health outcomes and its measurement is highly recommended in primary care settings. Functional performance and frailty are closely related concepts, and frailty status is associated with the use of health care services. However, there is insufficient evidence on the utilization of services profile according to the functional performance of older adults. The aim of this study was to assess the relationship between functional performance and the use of a wide range of health services in community-dwelling older adults. Generalized additive models for location, scale and shape were used to study these complex data of services utilization, from primary to hospital care. A total of 749 participants from two Spanish regions were followed up for 2 years. Of those, 276 (37%) presented low functional performance and 473 (63%) normal performance according to the Timed Up and Go test. The results showed that even after adjusting for burden of comorbidity and polypharmacy, participants with low functional performance used primary and secondary care health services more intensively, visited emergency rooms more often, and were hospitalized more frequently and for longer periods of time. A negative binomial distribution and a variant thereof were found to be the best models to describe health service utilization data. In conclusion, functionality should be considered as an important health indicator for tailoring the provision of health services for older adults.

## Introduction

Having a good functional ability is of great importance, and its preservation and promotion through the lifespan is a challenge for public health authorities and health systems [1]. Previous authors have suggested that comprehensive functional assessments in older people are significantly better predictors of survival and other health outcomes, compared to the presence of individual diseases or the amount of comorbidities [2]. The measurement of functional performance is recommended by the World Health Organization [1] and it could be easily implemented in primary health care settings [3, 4]. Thus, from a clinical perspective, approaches based on functional performance could be useful in framing public health responses and strategies towards a healthy ageing.

In this context, the evaluation and analysis of the health care services utilization of older adults, particularly of those with low functional performance, could help to explain the health trajectories of this population group and thus, optimize their care provision. Currently there is a lack of evidence in this area. Most published studies are focused mainly on frailty, or other factors, such us limitations in instrumental activities of daily living [5].

Several publications show an association between frailty and a more intensive use of health care services. In particular, it has been observed that frail people seek care at primary care providers and emergency rooms more often and are more likely to be readmitted to hospital after discharge than robust individuals [6–9]. Consequently, this utilization pattern is often associated with higher health care costs. Nevertheless, frailty is a complex concept that includes a miriade of deficits [10] and its measurement and cathegorization is not yet fully implemented at primary care settings [11].

Therefore, considering the role of functional performance as a part of the intrinsic capacity of aged persons and the existing recommendations for its use in all care settings [1], the aim of this study was to explore the use of a large range of health services in community-dwelling older adults, according to their functional performance. Due to the non-normally distributed nature of these kind of data, generalized additive models for location, scale and shape (GAMLSS) were used seeking to find the best distribution to fit the data.

## Materials and methods

### Study design

This research is based on a multicenter prospective cohort study, of community-dwelling older individuals, with a 2-year follow-up. Detailed information can be found in the study protocol [12].

### Participants

The target population was community-dwelling older adults aged 70 or over, who were functionally independent (Barthel Index >90 points [13]) from two primary care areas, Gipuzkoa and Costa del Sol, located on the northern and the southern coast of Spain, respectively. We excluded individuals who had a terminal illness, resided in a different area for more than 6 months a year and/or were unable to communicate in any of the local languages. Candidates willing to participate were asked to give written informed consent before inclusion. This study was approved by the corresponding Ethics Committees (CEIm Euskadi 01/2015; and CEI Costa del Sol 11Nov2014 PR Fragilidad).

### Data collection and variables

At baseline (May 2015 to July 2016), data were collected via face-to-face interviews performed by one trained nurse per site. Data on socio-demographic characteristics and lifestyle habits, like level of physical exercise, were collected. Health status variables were also assessed: active diseases, comorbidity by the age-adjusted Charlson Comorbidity Index (CCI), prescription drugs, and polypharmacy (>4 drugs). Functionality was measured using the 3-meter Timed Up and Go test (TUG). Individuals taking >12 seconds to complete the test were considered as having low functional performance [14]. Data of health resources use was obtained from the electronic health records of the participants: the number of contacts with the general practitioner and primary care nurse, in two different modalities, face-to-face and telephone consultations; visits to specialists; visits to emergency rooms; and hospital admissions and length of stay. Emergency room attendance and hospital admission were studied both as frequency of contacts, and as a binary variable, with categories “none” and “at least one”. Data on use of health resources was not available for one heatlh centre, so individuals from this centre were excluded from the present work.

### Statistical analysis

Categorical data are presented as frequencies and percentages (%) and quantitative variables as mean and standard deviation (SD), and also with medians and the first and third quartile (Q1, Q3), for non normally distributed quantitative data. Differences between participants according to their functional performance were assessed with the Chi-squared test for categorical data, Student´s t-test for quantitative data and the Wilcoxon rank-sum test for non normally distributed quantitative data.

GAMLSS models [15] were used to evaluate whether functional performance determined the use of health care services. Contacts with each specific service were included as counts in each model, and all models were adjusted for sex, polypharmacy, age-adjusted CCI, and the follow-up year (year 1 or 2). The site (Gipuzkoa or Costa del Sol) was included as a random effect. The follow-up time was considered to be the number of months between the date of one assessment (at baseline or year 1) and that of the following year’s assessment (at year 1 or 2, respectively), or the date of death, when this occurred before the next assessment.

As follow-up time could differ between participants, this was included in the models as an offset. GAMLSS allows modelling all the parameters of the distribution as functions of the explanatory variables. The parameters are: μ, the location parameter; σ, the scale parameter; and ν and ζ, the shape parameters, skewness and kurtosis, respectively. A GAMLSS model considers a wide range of distributions, continuous, discrete and mixed [16]. For each type of service use, the distribution that best fitted the data was chosen, based on the generalized Akaike information criterion (GAIC). Model selection was performed with a forward stepwise method using the stepGAIC function in the GAMLSS package [17]. The model results are reported as incidence rate ratios (IRR), and in the case of the logistic regression, as odds ratios (OR). Estimates are accompanied by their corresponding 95% confidence intervals (95% CI). Goodness of fit was studied by the worm-plot (a de-trended QQ-plot) of the residuals of each model. Ideally the points in the worm-plot should be close to the horizontal line in the middle with no systematic shape and 95% or more of the points inside the confidence brands. All analyses were performed with the free statistical software R, v.3.6.1.

## Results

At baseline, 754 individuals met the inclusion criteria, but 5 were excluded for having missing data on functional performance or health service use. Therefore, the present results are based on 749 participants, 58% from Gipuzkoa and 42% from Costa del Sol. Overall, 276 (37%) of the individuals presented low functional performance. Baseline characteristics of the sample are summarized in Table 1. Several differences were observed between groups. Participants with low functional performance were older, had lower income and poorer health-related characteristics than their robust peers. Additionally, a higher proportion of them were women.

**Table 1:** Baseline description of the participants overall and by functional performance.

	Total	Low	Normal	p-value
Sample size	749	276 (37%)	473 (63%)	
Age, *mean (SD)*	78.5 (4.8)	79.3 (5.3)	78.1 (4.5)	0.002
Sex *(female)*	401 (53%)	169 (61%)	232 (49%)	0.002
Level of education				0.110
Primary or less	598 (81%)	232 (85%)	366 (79%)	
Secondary	48 (6%)	14 (5%)	34 (7%)	
Higher	89 (12%)	26 (10%)	63 (14%)	
Low income *(≤€1*,*200/month)*	437 (62%)	185 (71%)	252 (56%)	<0.001
Living alone	317 (93%)	125 (93%)	192 (94%)	0.739
Smoking habits				0.021
Non-smoker	421 (57%)	249 (53%)	172 (63%)	
Former smoker	275 (37%)	190 (40%)	85 (31%)	
Smoker	45 (6%)	31 (7%)	14 (5%)	
Low physical activity level	85 (11%)	52 (19%)	33 (7%)	<0.001
Body mass index > 30 kg/m^2^	273 (36%)	118 (43%)	155 (33%)	0.007
Weight lost >4.5kg/year	53 (7.2%)	29 (6.2%)	24 (8.7%)	0.265
Self-perceived health status				<0.001
Good	559 (75%)	162 (59%)	397 (84%)	
Poor	190 (25%)	114 (41%)	76 (16%)	
Age-adjusted CCI, *mean (SD)*	4.4 (1.3)	4.6 (1.3)	4.3 (1.3)	0.002
Visual impairment	113 (15%)	54 (20%)	59 (12%)	0.012
Hearing impairment	151 (20%)	63 (23%)	88 (19%)	0.200
Falls in the last year	224 (30%)	106 (38%)	118 (25%)	<0.001
Polypharmacy *(>4 drugs)*	498 (67%)	209 (76%)	289 (61%)	<0.001

Data are presented as frequency (percentage), unless otherwise stated. SD, standard deviation; CCI, Charlson Comorbidity Index.

Data on health service utilization per follow-up year are shown in Table 2. Eight individuals were lost to follow-up during the first year; of these, seven died and one was institutionalized. Comparisons between groups indicated statistically significant differences in all the settings studied: primary and specialist care, emergency room attendances and hospitalization, services being used more frequently by participants with low functional performance than those with normal performance. Differences were more pronounced in the second year.

**Table 2:** Use of health services per year, by functional performance.

	Year 1			Year 2		
	Low	Normal	p-value	Low	Normal	p-value
***Sample size***	***276***	***473***		***272***	***469***	
**Primary care**						
GP (in total)	8.3 (7)	7 (5.7)	0.007	8.1 (7)	6.8 (5.3)	0.006
*median (Q1*,*Q3)*	6 (4,11)	6 (3,9)	0.015	7 (3,11)	6 (3,9)	0.047
Face-to-face	7.6 (6)	6.5 (5.1)	0.009	7 (5.4)	6.2 (4.8)	0.075
*median (Q1*,*Q3)*	6 (4,10)	5 (3,9)	0.021	6 (3,10)	5 (3,9)	0.118
Telephone	0.5 (1.5)	0.4 (1.1)	0.182	0.9 (2.6)	0.4 (1.3)	0.003
*median (Q1*,*Q3)*	0 (0,0)	0 (0,0)	0.890	0 (0,1)	0 (0,0)	0.004
PC nurse (in total)	5 (6.5)	3.3 (3.8)	<0.001	4.7 (5.4)	3.6 (10.9)	0.061
*median (Q1*,*Q3)*	4 (1,7)	2 (1,5)	<0.001	3 (1,6)	2 (0,4)	<0.001
Face-to-face	4.4 (4.8)	3 (3.6)	<0.001	3.7 (4)	3.2 (10.2)	0.341
*median (Q1*,*Q3)*	3 (1,6)	2 (1,4)	<0.001	3 (0.8,6)	2 (0,4)	<0.001
Telephone	0.2 (0.8)	0.1 (0.4)	0.051	0.5 (1.4)	0.3 (1.3)	0.072
*median (Q1*,*Q3)*	0 (0,0)	0 (0,0)	0.323	0 (0,0)	0 (0,0)	0.006
**Secondary care**						
Specialist (in total)	4.4 (4.3)	3.9 (4.3)	0.101	4.4 (4.5)	3.7 (4.3)	0.032
*median (Q1*,*Q3)*	3 (1,7)	2 (1,6)	0.011	3 (1,6.2)	3 (1,5)	0.002
Cardiologist	0.5 (1)	0.5 (1.3)	0.932	0.6 (1.3)	0.4 (0.9)	0.021
*median (Q1*,*Q3)*	0 (0,1)	0 (0,0)	0.259	0 (0,1)	0 (0,0)	0.015
Ophthalmologist	0.9 (1.8)	1 (1.8)	0.576	0.9 (1.7)	1 (2.2)	0.477
*median (Q1*,*Q3)*	0 (0,1)	0 (0,1)	0.292	0 (0,1)	0 (0,1)	0.605
Traumatologist	0.5 (1.2)	0.4 (1.1)	0.120	0.5 (1.1)	0.4 (1)	0.247
*median (Q1*,*Q3)*	0 (0,1)	0 (0,0)	0.011	0 (0,0)	0 (0,0)	0.169
Internist	0.3 (1.1)	0.1 (0.6)	0.014	0.3 (1.1)	0.2 (1)	0.111
*median (Q1*,*Q3)*	0 (0,0)	0 (0,0)	<0.001	0 (0,0)	0 (0,0)	0.001
**Emergency room**						
Attendance, *n (%)*	135 (49%)	200 (42%)	0.092	151 (55%)	204 (43%)	0.002
Emergency room	1.1 (1.7)	0.9 (1.6)	0.120	1.3 (1.9)	1 (1.6)	0.007
*median (Q1*,*Q3)*	0 (0,1)	0 (0,1)	0.060	1 (0,2)	0 (0,1)	0.001
**Hospital inpatient care**						
Hospitalization, *n (%)*	64 (23%)	96 (20%)	0.401	82 (30%)	95 (20%)	0.003
Hospital admissions	0.4 (0.9)	0.3 (0.8)	0.386	0.5 (1.2)	0.4 (1)	0.046
*median (Q1*,*Q3)*	0 (0,0)	0 (0,0)	0.315	0 (0,1)	0 (0,0)	0.003
Hospital stay *(in days)*	1.6 (6.2)	1.1 (5)	0.284	2.8 (12)	1.4 (7.5)	0.104
*median (Q1*,*Q3)*	0 (0,0)	0 (0,0)	0.159	0 (0,0)	0 (0,0)	0.035

The use of health services reflects the number of contacts per year, except for hospital stay data (which is in days). Data are presented as mean and standard deviation, unless otherwise stated. Binary variables (reporting the “at least one contact” category) are presented as frequencies and percentages, n (%); (Q1, Q3), first and third quartile; GP, general practitioner; PC, primary care.

Furthermore, these differences were still detectable in the multivariate GAMLSS adjusted models. The result of the best regression models, according to the GAIC criterion, showed that having low functional performance was significantly associated with utilization of every health service considered (Table 3). The negative binomial distribution type II was the distribution that best fitted most of the discrete outcomes, except for general practitioner visits for which the zero-inflated negative binomial distribution, type I, was chosen. The highest IRR was observed for the visits to a primary care nurse, indicating that individuals with low functional performance visited these nurses almost 50% more times over a year than those with normal performance. And they were also more likely to visit an emergency room or be hospitalized (30% and 40% more, respectively).

**Table 3:** GAMLSS models for the use of health services.

**Health service**	**General practitioner**	**Primary care nurse**	**Specialist**
	IRR (95% CI)	IRR (95% CI)	IRR (95% CI)
Intercept	0.38 (0.33,0.43)***	0.12 (0.10,0.14)***	0.27 (0.24,0.30)***
Functionality (*low*)	1.16 (1.07,0.54)***	1.47 (1.31,1.64)***	1.15 (1.03,1.28)*
Polypharmacy (*yes*)	1.41 (1.29,1.54)***	1.51 (1.34,1.69)***	1.49 (1.34,1.66)***
Age-adjusted CCI	1.06 (1.03,1.10)***	1.09 (1.05,1.13)***	---
Sex *(female)*	---	---	0.76 (0.70,0.84)***
Follow-up (*2*^*nd*^)	1.10 (1.02,1.19)*	---	1.12 (1.02,1.23)*
scale, σ	Sex^-^	Functionality^+^, Polypharmacy^+^, Follow-up^+^	Functionality^-^, Age-adjusted CCI^+^
skewness, ν	Functionality^+^	NA	NA
distribution	ZINBI (μ, σ, ν)	NBII (μ, σ)	NBII (μ, σ)
**Health service**	**Emergency room**	**Hospital admission**	**Hospital stay**
	OR (95% CI)	OR (95% CI)	IRR (95% CI)
Intercept	0.02 (0.02,0.03)***	0.01 (0.00,0.01)***	0.04 (0.02,0.07)***
Functionality (*low*)	1.31 (1.05,1.64)*	1.39 (1.07,1.80)*	1.36 (1.05,1.77)*
Polypharmacy (*yes*)	1.34 (1.06,1.71)*	1.54 (1.15,2.07)**	1.93 (1.38,2.69)***
Age-adjusted CCI	1.19 (1.09,1.29)***	1.18 (1.08,1.30)***	1.17 (1.07,1.28)***
Sex *(female)*	---	0.66 (0.51,0.85)**	0.56 (0.43,0.73)***
Follow-up (*2*^*nd*^)	1.29 (1.05,1.60)*	1.31 (1.02,1.68)*	1.36 (1.03,1.75)*
scale, σ	NA	NA	---
distribution	BI (n, σ)	BI (n, μ)	NBII (μ, σ)

The use of primary (general practitioner or nurse) and secondary care is described as number of contacts per year, length of hospital stay is measured in days, and emergency room attendance and hospital admission are dichotomous variables indicating none or at least one contact. For scale and skewness, the statistically significant variables are reported, indicating the sign of their coefficient as superindex.

IRR, incidence rate ratio; CI, confidence interval; OR, odds ratio; CCI, Charlson Comorbidity Index; ZINBI, Zero Inflated Negative Binomial type I; NBII, Negative Binomial type II; ---, variable not included in the final model; NA, not applicable for that distribution.

***p-value<0.001;

**p-value<0.01;

* p-value<0.05.

Functional performance was related not only to the location parameter but also to the scale parameter, regarding contacts with primary care nurses and specialists, and with the skewness, regarding contacts with general practitioners. These parameters influence the estimates of the mean and variance for each outcome (S1 Table).

Besides functional performance, polypharmacy and age-adjusted CCI were present in almost all models. Sex was only present in the use of specialists and hospitalization, and with coefficients smaller than 1, meaning that women used these services less than men. The period of assessment was also present in the models, indicating that the use of services increased during the second year of follow-up.

Contact frequencies or the likelihood of having contact with the different health services were also estimated by the GAMLSS models (Table 4). Estimates were calculated separately for participants with low and normal functional performance in our sample. Even after adjusting for other factors, compared to their robust peers, participants with low functional performance had more than one contact per year with their general practitioner, 8.5 (SD, 2.3) vs 7.1 (SD, 2.0), and also with their primary care nurse, 4.8 (SD, 1.4) vs 3.1 (SD, 1.0); and they, on average, stayed in hospital for half a day longer. Furthermore, the likelihood of visiting an emergency room was 50% and 40%, for individuals with low and normal functional performance, respectively.

**Table 4:** Estimates for usability per service by functional performance. Results from the GAMLSS models.

	Low	Normal
General practitioner	8.5 (2.3)	7.1 (2.0)
Primary care nurse	4.8 (1.4)	3.1 (1.0)
Specialist	4.4 (1.0)	3.8 (0.9)
Emergency room	0.5 (0.1)	0.4 (0.1)
Hospital admission	0.3 (0.1)	0.2 (0.1)
Hospital stay (days)	1.9 (1.1)	1.4 (0.9)

Data are presented as mean and the standard deviation. The use of primary (general practitioner or nurse) and secondary care (specialist) is reported as number of contacts per year, length of hospital stay is measured in days, and emergency room attendance and hospital admission are dichotomous variables indicating likelihood of having at least one contact with the corresponding service in a year.

**Fig 1:**
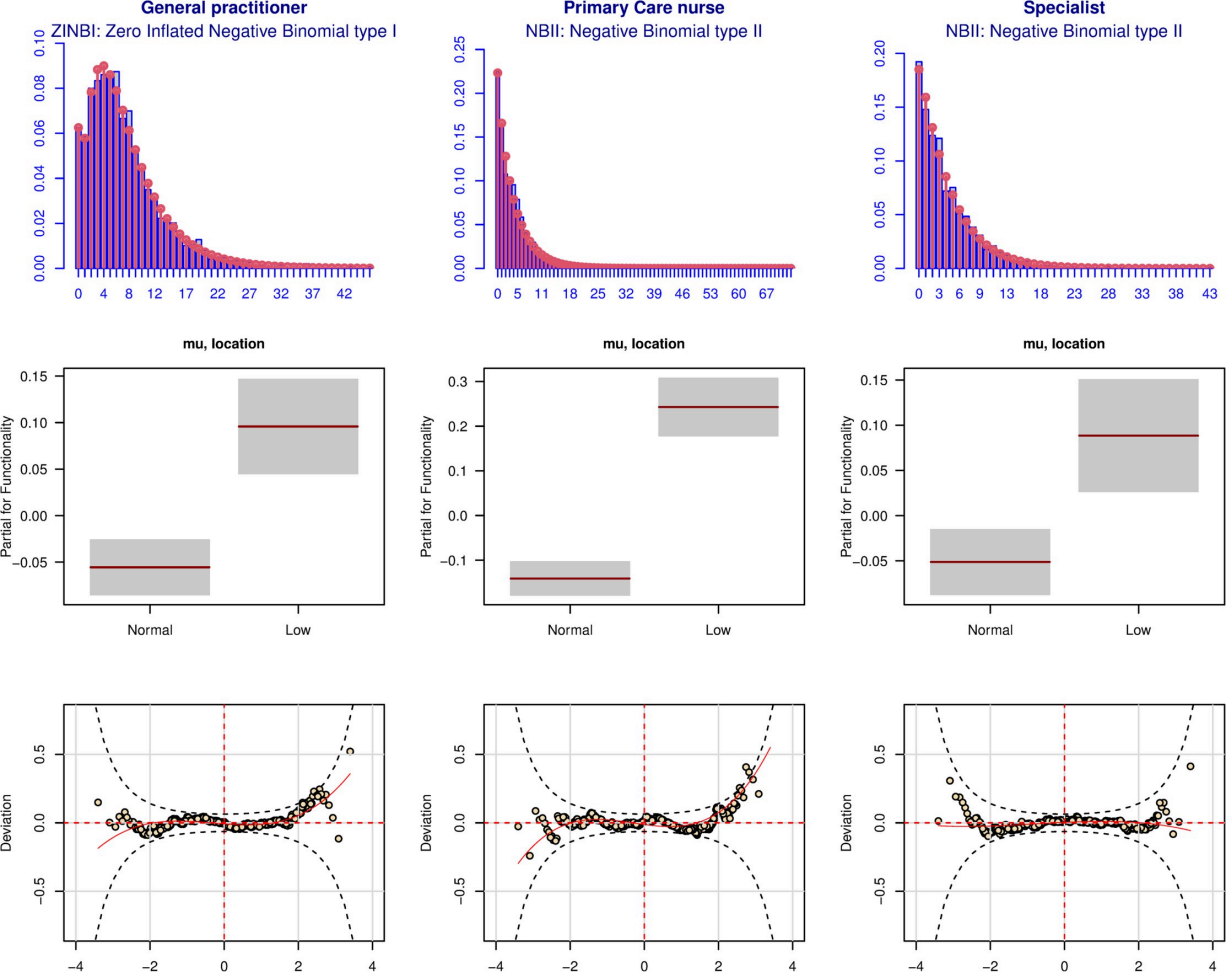
Goodness of fit of the models related to use of primary and secondary care services. The first row of figures shows the histograms of the observed data (in gray) where the selected distribution is adjusted (in red) for each service; second row, the partial effect of functionality in each model; and the third row, the worm-plots of the models (the more horizontal the red line, the better the model fits the data).

**Fig 2:**
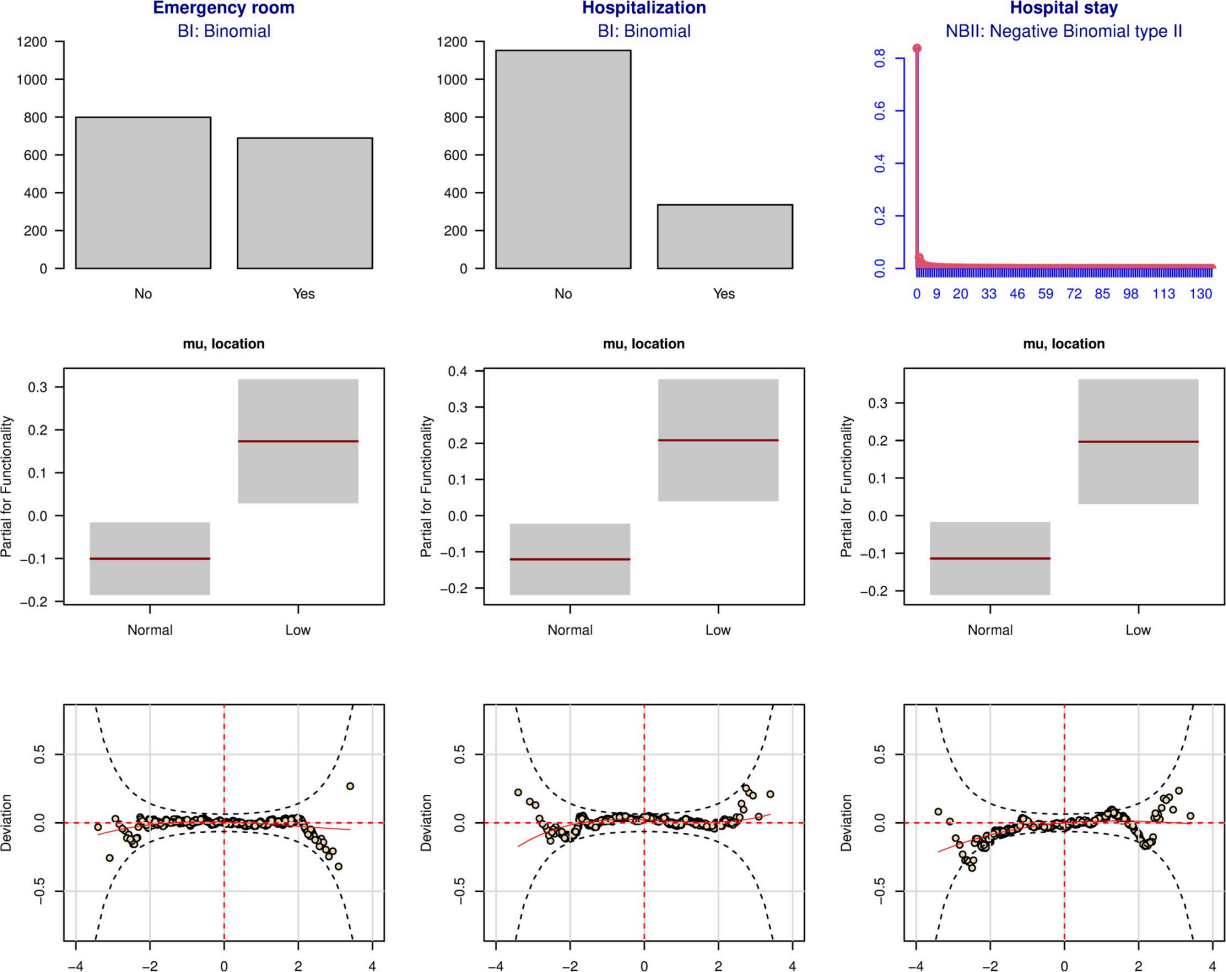
Goodness of fit of the models related to emergency room and hospital care. The first row of figures shows the histograms of the observed data (in gray) where the selected distribution is adjusted (in red) for each service; second row, the partial effect of functionality in each model; and the third row, the worm-plots of the models (the more horizontal the red line, the better the model fits the data).

The goodness of fit of the selected distribution and models for the use of primary and secondary care can be seen in Fig 1, and for the emergency room and hospital care in Fig 2. For these models, the distribution studied is plotted over the observed data. The partial effect of functionality is represented and the worm-plot of the model is also shown. Overall, the models fit the data well. Overall, the worm-plots were acceptable, as most of the points were inside the confidence brands.

## Discussion

We have studied the association between functional performance and the use of a wide range of health care services from primary care to hospital care over a 2-year follow-up study in community-dwelling older people. Knowing what kind of care services are most utilized by people with low functional performance could help in defining the most suitable care delivery routes for this population. Such knowledge would also help to estimate the potential effect of future interventions focused on maintaining functional performance.

The descriptive analysis shows a different profile in the use of health services between participants with low and normal functional performance. Functional performance could be related to certain socio-demographic characteristics and to the overall health status of an individual [18]. Therefore, it could be hypothesized that differences observed in the use of health care services are determined by the burden of morbidity, age, or demographic characteristics rather than by functional performance itself; however, the methodology used in this study allows for the assessment of the role of functionality adjusting for the aforementioned factors. Other studies in aged populations have also suggested that frailty and multimorbidity, rather than age, are both necessary to explain health care use [9] and the associated costs [19].

Functional performance, age-adjusted Charlson Index and polypharmacy explain most of the variation in the use of services observed in primary care, for both, general practitioners and nurses. Nevertheless, the burden of comorbidity as measured by Charlson index gains relevance in the models explaining the use of hospital services. Our results show that adults with low functional performance have one more consultation per year with their general practitioner and primary care nurse than their robust peers, and they stay in hospital for half a day more. They are also more likely to seek care through emergency rooms or be hospitalized. These results are in line with previous evidence concerning differences in health care utilization as a function of frailty. Therefore, following the WHO recommendations, it seems that the prevention of disease and the maintenance of functionality should become the main goals, in order to provide adequate health services to a patient [20]. Further, the TUG test could be considered an adequate tool to be used in consultations with primary care professionals [3, 4, 21].

The sex of the patient influenced the number of visits to specialists, number of hospital admissions and length of hospital stay. Women had lower rates of visits and hospitalizations than men. This is in line with the findings of a study investigating differences in health care utilization by gender in an older population in Spain [22], with similar results when adjusting the models for other health-related factors. These findings may imply inequalities in the provision of health care by gender, which need to be considered and tackled.

The differences found regarding health care utilization in this study are of great relevance, especially under adverse conditions, such as those of the current COVID-19 pandemic. Health care systems all around the world have had to implement essential changes due to the current situation [23]; with teleconsultations being offered instead of face-to-face visits with health care professionals and postponement of appointments with specialists and surgical procedures. All such changes have a huge impact on the health care delivered and could result in a widening of existing gaps. This makes it even more important to identify differences in the delivery of health care in order to be able to adjust and plan the provision of care to vulnerable groups.

The present study has several strengths. It adds to the scarce existing evidence, by providing knowledge on the utilization profile of older adults with low functional performance on a large range of health care services. Another strength, and at the same time a novelty in this setting, is the use of GAMLSS models. These models are capable of handling non-normally distributed outcomes, searching for the best distribution that fits the data, and providing robust estimates. They are well suited to health care utilization data, which is mainly zero-inflated and skewed, and can help to achieve a more appropriate analysis of these outcomes than that obtained with other approaches. Presenting these models here may encourage others to explore similar outcomes more appropriately. Previous articles have also applied the negative binomial distribution type I to study the use of health services among frail persons [9, 24, 25]. In our case, the results suggested that the distribution that best fit our data were the variants of the negative binomial, negative binomial type II, and zero-inflated negative binomial type I. Moreover, these models have shown that functionality seems to be related not only to the location of the distribution, but also to the scale and shape, meaning that data from individuals with low and normal functional performance have distinct characteristics, with different variances, for example.

The main limitation of the study is that the sample is probably not entirely representative, as willingness to participate may be related to a person’s health status, with those in better health being more likely to agree. Certain studies have indicated that automated variable selection methods could include non-truly significant variables in a model. This probability increases as the number of the initially considered variables increases [26]. In our case, the pre-selection of the 5 initial variables was based on clinical expertise. Given their low number and their clinical relevance, the above mentioned risk appears to be rather reduced in this work.

## Conclusion

Low functional performance is associated to a more intense use of health care resources. The present findings suggest that other currently used health indicators, like the burden of the disease and the number of prescribed drugs alone, do not suffice in predicting the provision of health care services for order people. Functional performance could play a key role as a relevant health indicator, beyond frailty. As such, it has the potential to improve the prediction of health care services demands and to adapt the clinical management of older people to their needs. Its evaluation at the primary care level is an asset, which enhances its value.

## Supporting information

S1 Table Detailed information about GAMLSS models for each health care service. μ, location; σ, scale; ν, skewness; Functionality measured by Time Up-and-Go test; CCI_adj_, age-adjusted Charlson Comorbidity Index; ZINBI, Zero Inflated Negative Binomial type I; NBII, Negative Binomial type II; BI, Binomial.
(DOCX)
