# Supplementary material for: Use of health care services according to functional performance in community-dwelling older adults in Spain. An approach using GAMLSS models
Source: PLoS One. 2022 Nov 17;17(11):e0277681. doi: 10.1371/journal.pone.0277681 (PMC9671440; doi:10.1371/journal.pone.0277681)
Supplement: S1 Table — μ, location; σ, scale; ν, skewness; Functionality measured by Time Up-and-Go test; CCIadj, age-adjusted Charlson Comorbidity Index; ZINBI, Zero Inflated Negative Binomial type I; NBII, Negative Binomial type II; BI, Binomial. (DOCX) [file pone.0277681.s001.docx]

**S1 Table. Detailed information about GAMLSS models for each health care service.** µ, location; σ, scale; ν, skewness; Functionality measured by Time Up-and-Go test; CCI_adj_, age-adjusted Charlson Comorbidity Index; ZINBI, Zero Inflated Negative Binomial type I; NBII, Negative Binomial type II; BI, Binomial.

| **General practitioner** |
| --- |
| Distribution: ZINBI (*µ, σ, ν*) |
| Estimated expression of the parameters:  $\log\left( \hat{\mu} \right)=-0.98+0.15 Functionality+0.35Polypharmacy+0.06{CCI}_{adj}+0.10 Period2$  $\log\left( \hat{\sigma} \right)=-0.81-0.24Female$  $logit \left( \hat{\nu} \right)=-4.20+1.14Functionality$ |
| Mean and variance:  $E\left( Y \right)=\left( 1-\nu\right)\mu$  $Var\left( Y \right)=\left( 1-\nu\right)\mu+ (1-\nu)(\sigma+\nu)\mu^{2}$ |
| **Primary care nurse** |
| Distribution: NBII (*µ, σ*) |
| Estimated expression of the parameters:  $\log\left( \hat{\mu} \right)=-2.10+0.38 Functionality+0.41Polypharmacy+0.09{CCI}_{adj}$  $\log\left( \hat{\sigma} \right)=0.36+0.44Female+0.64Polypharmacy+0.29Period2$ |
| Mean and variance:  $E \left( Y \right)=\mu$  $Var\left( Y \right)=\mu+\sigma\mu$ |
| **Specialist** |
| Distribution: NBII (*µ, σ*) |
| $\log\left( \hat{\mu} \right)=-1.33+0.14 Functionality+0.40Polypharmacy-0.27Female+0.11Period2$  $\log\left( \hat{\sigma} \right)=1.10-0.19Functionality+0.04{CCI}_{adj}$ |
| Mean and variance:  $E \left( Y \right)=\mu$  $Var\left( Y \right)=\mu+\sigma\mu$ |
| **Emergency room attendance** |
| Distribution: BI (*n, σ*) |
| Estimated expression of the parameters:  $\mathrm{logit} \left( \hat{\mu} \right)=-3.79+0.27 Functionality+0.30Polypharmacy+0.17{CCI}_{adj}+0.26Period2$ |
| Mean and variance:  $E \left( Y \right)=n\mu$  $Var\left( Y \right)=n\mu(1-\mu)$ |
| **Hospitalization** |
| Distribution: BI (*n, µ*) |
| Estimated expression of the parameters:  $\mathrm{logit} \left( \hat{\mu} \right)=-4.80+0.27 Functionality+0.43Polypharmacy+0.17{CCI}_{adj}-0.42Female+0.27Period2$ |
| Mean and variance:  $E \left( Y \right)=n\mu$  $Var\left( Y \right)=n\mu(1-\mu)$ |
| **Hospital stay** |
| Distribution: NBII (*µ, σ*) |
| Estimated expression of the parameters:  $\log\left( \hat{\mu} \right)=-3.25+0.31 Functionality+0.66Polypharmacy+0.16{CCI}_{adj}-0.59Female+0.31Period2$  $\log\left( \hat{\sigma} \right)=3.39$ |
| Mean and variance:  $E \left( Y \right)=\mu$  $Var\left( Y \right)=\mu+\sigma\mu$ |
